# Promoting Racial Justice in Cancer Clinical Trials: Community Engaged Solutions for Bridging Gaps

**DOI:** 10.1002/cam4.70690

**Published:** 2025-02-19

**Authors:** Deana M. Williams, Chaya M. Pflugeisen, Khadijah Ameen, Toby K. Chen, Sheree Cooks, Bernadette C. Ray, Melissa Foster, De Phillips, Lizbeth Sanchez, Keywanna Hardiman, Cynthia Smith, Jennifer Slim

**Affiliations:** ^1^ MultiCare Health System Institute for Research & Innovation Puyallup Washington USA; ^2^ Clinical Trials Advisory Group Puyallup Washington USA; ^3^ MultiCare Cancer Institute Tacoma Washington USA

**Keywords:** clinical trials, health equity, healthcare, inclusion, oncology, racial and ethnic diversity, racial justice

## Abstract

**Introduction:**

Racially and ethnically diverse populations are disproportionately burdened with and more likely to die from cancer than White populations but remain persistently underrepresented in cancer clinical trials. Thus, the safety and efficacy assessments of novel therapies may not apply to all populations, and guidelines are developed using trials that inadequately reflect our population. This study aims to explore healthcare experiences, cancer clinical trial perceptions, and solutions for bridging gaps in cancer clinical trials among communities of color.

**Methods:**

We held a series of five focus groups with 23 survivors and caregivers of color. These sessions explored perspectives around cancer care and research, clinical trials recruitment and messaging, and priorities around addressing the current insufficiency in representation in cancer research.

**Results:**

Our focus group data was analyzed using an inductive thematic approach. Four themes were present including (1) high motivation to participate in cancer trials and desire for better outreach and education within communities of color; (2) examples of just care as a model for improving access to clinical trials; (3) manifestations of power and inequity in healthcare inhibiting opportunities for clinical trial participation; and (4) the need for trust‐building at the healthcare, community, and interpersonal levels.

**Conclusion:**

Our findings demonstrate that people of color strongly desire clinical trial options and discussions during cancer care, and trust‐building is foundational to opening pathways to research participation. We offer concrete steps for increasing racial justice in cancer clinical trials that serve as a point of reflection and guidance for healthcare professionals, researchers, and clinical trials teams.

## Abstract 2

Cancer patients of color want clinical trial options and improved efforts to engender trustworthiness within healthcare and medical research. We offer tangible, community‐engaged, anti‐racist approaches to advancing such efforts.

## 1 Introduction

Cancer is the second leading cause of death in the US [1, 2]. Racially and ethnically diverse populations are disproportionately burdened and more likely to die than White populations from multiple cancer types [2, 3, 4]. In 1993, the National Institutes of Health mandated the inclusion of women and ‘minorities’ in federally funded research [5], but three decades later, the proportion of racially and ethnically diverse patients enrolled in clinical trials remains alarmingly low [6]. From 2010 to 2016, cancer clinical trials seeking FDA approval overwhelmingly included White participants (82.3%) [7]. In 2020, people of color comprised more than 40% of the US population [8], but only 25% of participants in FDA‐approved clinical trials for diseases including cancer [9].

Because racial and ethnic subgroups have different experiences and exposures, they may respond to medications differently [10]. The persistent lack of inclusion of communities of color within cancer trials means research findings may not apply to all populations [11], leading to inadequate understandings of the safety and efficacy of novel therapies in racially and ethnically diverse populations [12]. Cancer trials also shape screening and clinical decision‐making guidelines, meaning recommendations have been developed based on trials that are not reflective of communities of color [13].

Although barriers to racial and ethnic diversity in clinical trials are well documented [14, 15, 16, 17, 18], developing strategies for removing these barriers remains a significant challenge [19]. Strategies are often developed without community partnership and many lack an anti‐racist focus, despite the historic and contemporary entrenchment of racism within healthcare and clinical research. Community engagement has long been recognized as a critical element of equitable research [20, 21, 22], and calls for community engagement in cancer trials are increasing [23, 24, 25]. Recent declarations of racism as a public health threat in the United States [26, 27] and new legislation requiring clinical trial diversity plans [28] have called upon cancer researchers to address structural racism in their institutions and studies [29] in an effort to mitigate racial and ethnic disparities in cancer outcomes. But there remains an urgent need for more community‐oriented, anti‐racist approaches to increasing representation for communities of color in clinical trials, which can lead to lasting and scalable changes [19, 30, 31].

Responding to this need, this study used anti‐racist research principles [32, 33] (Table 1A) and community engagement to hold focus groups exploring healthcare experiences, cancer clinical trial perceptions, and community‐endorsed solutions for bridging gaps in cancer clinical trials. In this manuscript, we share findings from these focus groups, applications of anti‐racist research principles (Table 1B), and tangible actions to promote racial justice in cancer clinical trials (Table 1C).

**TABLE 1:** (A–C) Anti‐racist research principles, application, and actions.

(A) Research Principle	(B) Application within this study	(C) Actions to improve clinical trial access
1. Center the voices of communities of color.	Focus group participants were exclusively patients and caregivers of color.	Involve communities of color in developing culturally competent materials to improve outreach and education initiatives. Seek to understand what matters most to patients and whether available clinical trials best serves those priorities.
2. Engage community members as partners in the research process to create change.	Focus group guide co‐development with Community Advisory Group. Focus group co‐facilitation with CAG member. Shared study decision‐making with CAG.	Involve communities of color in developing culturally competent materials to improve outreach and education initiatives.
3. Use interdisciplinary teams to bring multiple perspectives to the project.	Collaboration with: CAG members who hold professional roles in oncology and community institutions. Cancer Institute staff. Cancer research conduct staff.	Intentionally engage and convene diverse oncology professionals across all healthcare system levels, areas (admin, prevention, treatment, research), and sectors in efforts to improve clinical trials representation.
4. Practice cultural humility.	Shared decision‐making with CAG members from multiple communities. Intentional reflexivity journaling prior to and following focus groups and during analysis.	Involve communities of color in developing and refining clinical trial messaging to ensure materials are responsive to cultural values, linguistic needs, and intersecting identities. Dedicate person and financial resources for healthcare and trial staff to unlearn bias. Seek to understand what matters most to patients in a clinical trial, dedicating space and time to discuss questions and priorities.
5. Engage in self‐reflection to maintain awareness of one's perspectives, biases, and privileges.	Intentional reflexivity journaling prior to and following focus groups and during analysis. Team reflexivity conversations throughout the project lifecycle.	Dedicate person and financial resources for healthcare and trial staff to unlearn bias.
6. Collect qualitative research to understand lived experiences.	Focus groups designed to elicit input on intervention.	Hold listening sessions, town halls, and develop community advisory boards dedicated to understanding community perspectives and needs and experiences around and within cancer clinical trials.
7. Prioritize empowerment and research benefit for communities over scientific colonization.	Intervention development *following* focus groups to ensure community priorities and perspectives drive the effort.	Discuss clinical trials with all patients of color, regardless of eligibility status. Expand availability of decentralized trials to provide patients of color more control over where and how they participate.
8. Seek to dismantle factors that contribute to inequities.	Offer focus groups virtually, including evening and Saturday sessions. Compensate participants equitably for their time.	Concrete steps to diversify the healthcare and clinical trials workforce: Engage and sustain students of color in medical fields. Increase mentorship and networking opportunities. Support Principal Investigators of color. Improve hiring and retention of healthcare professionals from minoritized communities. Expect White healthcare professionals to invest and engage in antiracism efforts. Dedicate staff, optimize workflows, and increase budgets to allow for holding discussions about clinical trials with all patients of color.
9. Explicitly examine racism within data analysis.	Qualitative analysis through anti‐racist lens. Naming racism in participant stories.	Examine trends related to clinical trial enrollment within your institution and embed anti‐racist principles to address gaps.
10. Disseminate findings to all communities who will benefit from the research.	Focus group findings shared back to community through multiple engagement efforts.	Ensure clinical trials participants receive findings demonstrating the impact and outcomes of their participation.

## 2 Materials & Methods

### 2.1 Theoretical Framework

We used an Anti‐racist Framework to guide our study. Anti‐racism is the intentional practice of opposing and dismantling racism by creating policies, practices, systems, and structures that lead to racial equity [34]. Thus, anti‐racist research is an action‐oriented paradigm that seeks to illuminate and disrupt racism through equity‐based research methods [32]. Central to this paradigm is the notion that anti‐racist practices must be embedded within all aspects of the research process. While literature on the necessity of anti‐racism within the health sciences and medicine is increasing, fewer studies have proposed anti‐racist research frameworks or principles that offer strategies for integrating anti‐racist practices across the entire project lifecycle. In our literature review, we identified two frameworks [32, 33] and one toolkit [35] that provided in‐depth recommendations for incorporating anti‐racism into research practices from study design through dissemination. We consolidated each set of principles and recommendations to identify patterns across all three resources, leaving us with 21 principles. Finally, we removed any principles that were not relevant to our study, such as recommendations related to machine learning. The final ten principles that we integrated into our study can be found in Table 1A.

### 2.2 Participants and Recruitment

We began by establishing a racially and ethnically diverse Community Advisory Group (CAG), recruiting advisory group members through outreach to community‐based organizations, professional and personal networks, and social media. The Principal Investigators met virtually with 15 of 21 people interested in joining the CAG to discuss the opportunity and the potential participants motivation and experience. The final nine‐person committee includes cancer survivors and caregivers, community leaders, and oncology healthcare professionals. Five CAG members are Black, one is Chinese, one is Inupiaq, one is Latinx, and one is White; all but two are women.

We recruited focus group participants through our healthcare system, regional organizations, and professional networks. To be eligible, participants needed to be a person of color; a survivor/caregiver of breast, colorectal, lung, or prostate cancer; and not have participated in a cancer clinical trial. Because we were focused on understanding the perspectives and priorities of people who had not participated in a clinical trial, we specifically recruited individuals who had navigated cancer care without clinical trial enrollment. Forty‐four individuals expressed interest in participating by completing an online intake form that both screened for inclusion criteria and collected relevant demographic data. Each eligible participant was then offered the opportunity to review and complete the consent form using the REDCap [36] e‐consent framework and/or speak with a member of the study team if they had questions or sought support in the consent process. During the consenting process, participants were asked to select pseudonyms to protect their identity. Focus group attendees were compensated $100 for participation. Ethics approval for this study was obtained by the Institutional Review Board at the Principal Investigators' Institution.

### 2.3 Data Collection and Analysis

We worked with our CAG to co‐develop a semi‐structured focus group guide informed by relevant literature and collective expertise (Table 2). In February 2024, study team dyads (DW/CP, DW/KA), all of whom had personal and/or professional experiences relevant to cancer equity, facilitated five focus groups. All dyad members also had previous training and experience leading qualitative focus groups. Each 2‐h session was held and audio/video recorded using our institution's HIPAA‐compliant virtual meeting software (Microsoft Teams). After each session, verbatim transcripts were produced using a professional transcription service.

**TABLE 2:** Sample of focus group guide questions.

Focus group topic	Sample focus group question
Perceptions and knowledge of cancer clinical trials	What do you think of when someone refers to cancer research or cancer clinical trials? What types of conversations or messages have you heard about cancer clinical trials from the people in your family, community, or the media?
Experiences with the healthcare system prior to cancer diagnosis	Before you or your loved one was diagnosed with cancer, how did you or your loved one interact with the healthcare system? What were those experiences with the healthcare system like?
Experiences with racism in healthcare	How has racism, or forms of racial injustice, impacted you or your loved one's experience of cancer treatment?
Decision‐making considerations around clinical trial enrollment	If you or your loved one *were* offered a clinical trial, but decided not to enroll, can you tell us about what shaped this decision not to participate? If you or your loved one *were not* offered a clinical trial, do you imagine that you or your loved one would have considered participating if the opportunity was there? Why or why not?
Perceptions around healthcare system trust‐building	To what degree do you trust the providers and healthcare systems that offer clinical trials, or that clinical trials might benefit communities of color?
Desires for bridging the gap between cancer patients from communities of color and cancer clinical trials	What would need to happen for you to trust the providers and healthcare systems that offer clinical trials, or to trust that clinical trials might benefit communities of color? Imagine a person who works for the healthcare system and helps enroll people from communities of color on cancer clinical trials. What characteristics would you want that person to have? What role could that person play in helping you or your loved one enroll on and navigate a clinical trial?

Our lead researchers (DW, CP) developed an analytic codebook within Dedoose qualitative data management software. Though most codes were formed inductively, select a priori codes (e.g., racism, health inequity, cultural humility) were established based on the focus‐group guide and the anti‐racism principles guiding our work. We analyzed the data using the five steps of thematic analysis outlined by Braun & Clarke [37], a well‐established qualitative analytic approach that supports inductive and deductive coding [38]. We maintained an audit trail, tracking codebook progressions and revisions, and held weekly meetings to adjust the codebook, discuss emergent data patterns, and share reflexive journal writings. These meetings helped us remain engaged with the data, collectively establish decision‐making, assess coding consistency, and remain rooted in racial justice principles.

## 3 Results

Twenty‐three individuals participated across five focus groups, and each focus group hosted 4–6 survivors and/or caregivers. Participants ranged in age from 29 to 73 and claimed multiple racial, ethnic, and gender identities (Table 3). None of our participants or their loved ones had participated in a cancer clinical trial. Our analysis generated four themes, described in detail below.

**TABLE 3:** Focus group participant demographics.

Pseudonym	Survivor/caregiver	Cancer site	Treatment state	Race/ethnicity	Gender	Age
Jei	Survivor	Breast	WA	Afro Indigenous	Two Spirit	29
Michelle	Survivor	Breast	WA, HI	Mexican and Caucasian	Female	34
Ivanna	Survivor	Breast	WA	Cambodian/White	Female	37
Sydney	Survivor	Prostate	WA	Black Caribbean	Male	73
Pat	Survivor	Breast	TX, WA	Black	Female	55
Betty	Survivor	Breast	WA	African	Female	64
AJ	Survivor	Breast	WA	Asian	Female	68
Anne	Survivor	Lung	WA	Black	Female	68
Simon	Survivor	Prostate	NY	Black Latino	Male	70
Jessica	Survivor	Breast	WA	Black	Female	38
My Mother's Footsteps	Survivor & Caregiver	Breast	WA	African Indigenous	Female	47
Dana	Survivor	Breast	WA	Black	Female	51
Elizabeth	Survivor	Breast	WA	Hispanic	Female	51
Laurie	Survivor	Breast	WA	Hispanic	Female	67
John	Survivor & Caregiver	Prostate	WA	Black/Native American	Male	62
Antoinette	Survivor	Breast	WA, GA	Black	Female	47
Patty	Survivor & Caregiver	Breast	WA	Asian	Female	58
Mimi	Caregiver	Prostate	GA	African American	Female	28
Marvin	Survivor	Prostate	WA	Black	Male	62
Singer	Survivor & Caregiver	Breast	WA	Black ‐ Caribbean/Afro‐Latina	Female	42
Mary	Caregiver	Breast	WA	Black	Female	45
Marie	Survivor	Breast	WA	Native American/Latina	Two Spirit	66
Jasper	Survivor	Lung	WA	Black and White	Transgender Male	37

### 3.1 Motivations & Desires Shaping Clinical Trials Decision Making

When asked if they would have participated in a clinical trial if given the opportunity, nearly all participants responded “yes.” Participants believed clinical trials could improve their quality of life and treatment experience. Jei would have participated in a trial that was less lengthy and burdensome than their course of treatment.

I would've taken the opportunity had I been offered, especially if it seemed less strenuous than the six rounds of chemo, 30 rounds of radiation, and five years of hormone suppression that they prescribed.

Mimi felt her grandfather would have selected a trial if it offered a greater chance of survival.

[My grandfather] would want to know the pros and cons. I think he would pick whichever [treatment] gave him the best fighting chance. And if it happened to be the clinical trial, I think he would go with it.

Altruism also motivated trial acceptability. Participants noted that trials improve research and care benefiting others, recognizing that racially and ethnically diverse participants are persistently underrepresented within cancer studies. Antoinette spoke of her strong desire to participate as a way to give back, saying, “I want to help, I want to be a beacon. With the life I've been given, I wanna make sure that I'm a voice [for] caregivers and survivors.

Others felt clinical trials provided opportunities to receive cutting‐edge treatments they otherwise would not be able to access, and some participants proactively sought clinical trial opportunities for this reason. Elizabeth was trying to enroll in a trial at the time of the focus group in hopes of improving her long‐term outcomes:

I had triple‐negative breast cancer, and there's clinical trials going on for a vaccine that I would like to be part of. My cancer has a high reoccurrence rate, so, it's the first glimmer of hope within five years.

Finally, participants desired increased transparency surrounding clinical trials. These discussions largely stemmed from concerns around gatekeeping, underscoring the power dynamics within healthcare systems that often limit patient agency. Participants wanted to be notified not only if a trial was available, but even if no trials were available or if they were found to be ineligible for available trials. Simon had attempted to enroll in a trial, completing an online application at three different sites, but received no follow‐up. Ivanna captured this desire, saying:

Address whether there are clinical trials available or not because it wasn't talked about and throughout my whole experience, it made me assume that there wasn't anything available.

Both Simon and Ivanna's experiences emphasize how direct communication surrounding clinical trials would have provided more clarity about their treatment options and created an opportunity for trust‐building with their care team, regardless of their participation eligibility.

### 3.2 Foundations of Just Care as a Model for Clinical Trials

When discussing healthcare interactions before and after their cancer diagnoses, many participants recounted justice‐oriented patient‐centered experiences. They described being “treated with compassion and cultural humility,” “believed,” “reassured,” and “listened to” by their care teams. It has been shown that trusting relationships with healthcare professionals and culturally grounded care practices influence clinical trial enrollment and retention [39], making patients' past and current care experiences influential in their willingness to participate in clinical trials. Accordingly, participants' deeply resonant, equitable healthcare experiences illuminate justice‐based behaviors and practices that can be strived for within clinical trials to improve racial and ethnic representation.

Many participants voiced an “unspoken comfort” with their cancer care team members who reflected their identities or cultural backgrounds, like Marie, who shared:

Everybody through [the system where I received care], they have all been women of color, which was amazing to me…I didn't even ask for women of color, it just happened and that made me very comfortable.

The sense of connection and safety with healthcare professionals who shared their identities, articulated by Marie and others (Table 4), underscores the importance of building and sustaining a diverse workforce among clinical trials sites.

**TABLE 4:** Themes and Representative Quotes.

Themes	Representative Quotes
Motivations & Desires Shaping Clinical Trials Decision Making	“I tell my doctors, let me stick around for as long as I can, you know. Any little bit will helps if a trial comes up that will help me.” John, Black/Native American Prostate Cancer Survivor and Caregiver, age 62 “I would have participated like 100%. I just, yeah, I think that it's important to contribute to the body of knowledge for cancer treatment.” Elizabeth, Hispanic Breast Cancer Survivor, age 51 “I think of [clinical trials] as a way of expanding what is currently out there [and] something that is gonna help in the future.” Jasper, Black & White Lung Cancer Survivor, age 37 “If somebody was to take their time to answer all my questions with empathy, then I know that'll make me want to do a clinical study.” Mary, Black Caregiver of a Breast Cancer Survivor, age 45 “I would wanna have all the information, that way you could take it home. And scheduling extra time at the next appointment gives you time to read the paperwork and come up with questions.” Pat, Black Breast Cancer Survivor, age 55
Foundations of Just Care as a Model for Clinical Trials	“I felt more comfortable when I was back home in Hawaii, getting my cancer treatment with the care team that was Filipino or mixed ethnicities. It just felt like the communication styles were similar, there was an unspoken comfort with one another.” Michelle, Mexican and Caucasian Breast Cancer Survivor, age 34 “This was a young White physician. He just pulled up his bench, came right to eye level. He didn't look down on me, he met me right where I was.” Antionette, Black Breast Cancer Survivor, age 47 “He is very compassionate. He is very kind. [It] feels like he's listening, and he's with you in the moment.” Jessica, Black Breast Cancer Survivor, age 38, referring to her provider “I went to the doctor regularly before my diagnosis…My GP I've been with since 2019, and I think that relationship building kind of helped in them taking me seriously.” Jei, Afro Indigenous Breast Cancer Survivor, age 29
Manifestations of Power & Inequity in Healthcare	“Black and brown people are more apt to die of cancer than White people and we all know that. And we all know why.” Anne, Black Lung Cancer Survivor, age 68 “My physician didn't think anything was wrong and had to keep advocating and going back in order to get an appointment. I still had to wait 2 months after, so the cancer was grade three and stage three when I got diagnosed.” Jessica, Black Breast Cancer Survivor, age 38 “I didn't have insurance…the folks used to walk around with a paper with my name on it and on the back of the paper in big blue letters was **UNINSURED**. So that's how I was labeled.” My Mother's Footsteps, African Indigenous Breast Cancer Survivor, Age 47
Multi‐level Approaches for Trust‐Building	“Have representation there, not paid actors, who will talk about clinical trials and the positive results for people of color. Show what it may help with. Have them be open, honest, sincere, gracious, and really talk about everything. And then I think you would probably get a lot more people of color, people in the LGBTQ community being a part of this, if they go, oh, that person's like me.” Marie, Native American & Latina Breast Cancer Survivor, age 66 “I would prefer it to be someone that is not overwhelmed. I would want someone that has the opportunity to really get to know me or doesn't have so much on their plate that they can't even deal with things themselves.” My Mother's Footsteps, African Indigenous Breast Cancer Survivor, age 47, on her desires for a healthcare professional who could bridge gaps to clinical trials “Following up, not just coming once. [Cancer is] so all‐consuming and overwhelming. [They need to] follow up when you've been able to catch your breath a little bit.” Laurie, Hispanic Breast Cancer Survivor, age 67, on her desires for a healthcare professional who could bridge gaps to clinical trials

While experiences with racially/ethnically/culturally concordant healthcare professionals were overwhelmingly described as positive, concordance was not the defining factor in establishing trusting relationships. Participants described ways that some White providers conveyed trustworthiness and compassion through their actions and attitudes.

I have had many healthcare providers who are White who have treated me with dignity, with respect. They've shown concern for my health and well‐being to an extent that made me feel confident that I was being looked after in the best way possible.

Simon's narrative transcends applicability to primary care by depicting qualities of trustworthiness in White providers.

Finally, patient/participant agency was discussed as a component of just care. Elizabeth provided an example of this, saying:

I opted for a double mastectomy, even though the protocol asked for a lumpectomy, and [my breast surgeon] was like It's your body, I will do whatever you feel comfortable with.

### 3.3 Manifestations of Power and Inequity in Healthcare

Focus group participants called attention to the overlapping structures of power and oppression entrenched within healthcare that perpetuate health inequity and cancer clinical trial disparities among communities of color. Highlighting these experiences provides important context for understanding how they might insidiously contribute to the exclusion of communities of color in clinical trials. First, participants experienced dismissal and disbelief of their cancer concerns. For instance, Laurie's narrative exemplifies how the intersections of sexism and racism contribute to women of color's cancer concerns being minimized by providers.

Being a woman of color, you have so many obstacles and hoops you have to jump through. Doctors just don't take [you] seriously when [you] know [your] own body.

Laurie's experience rang true for multiple participants who discussed fighting to receive a cancer screening, like Pat who said her providers “gave me all their reasons [why I couldn't get a mammogram] and I gave them all the reasons why [I could]… And that's how I found my stage one cancer.” Early detection of cancer provides the best chance for successful treatment but also allows patients to be connected clinical trials. Delayed diagnoses, which disproportionately impact communities of color and Black communities in particular [40, 41, 42], limit opportunities for participation in trials.

Participants also experienced disrespect and mistreatment. Jasper, whose cancer was diagnosed incidentally following an injury said, “I didn't really go to doctor's appointments … I've had better experiences with going urgent care, and that's what I've always done.” For Jasper, healthcare avoidance is a self‐protective measure driven by previous experiences with racism, weight stigma, and anti‐transgender attitudes within primary care. Thus, mistreatment and disrespect can also lead to diagnosis delays. My Mother's Footsteps brought individuals from her social support network to her cancer treatment appointments, “so that they could protect me” from racist treatment from her radiation team. Other self‐protective measures discussed by participants included code‐switching, dressing up for appointments to appear “presentable”, and altering aspects of their appearance such as covering tattoos and removing piercings. Singer described being cognizant of her demeanor and language in front of her providers, saying:

At the end of the day, I have to make sure that I code‐switch, so that I can make sure that I get the best care. I tried to do my best under these terrible circumstances to try and ameliorate as much of any potential racism that I would face.

These examples demonstrate why efforts to reduce racial and ethnic gaps in clinical trials require a purposeful commitment to remedying structural oppression, not just within cancer care, but across all facets of the healthcare system.

### 3.4 Multi‐Level Approaches for Trust‐Building

Participants cited trust as foundational to clinical trials participation and offered culturally grounded, community‐engaged trust‐building approaches spanning healthcare, community, and interpersonal levels. Participants like Simon proposed intentional healthcare system efforts focused on repairing relationships with communities of color and demonstrating anti‐racist accountability, such as outreach that explicitly acknowledges the current insufficiency of representation in clinical trials.

I want to see something in writing that said, ‘we are reaching out to folks who have been underserved because we would like to expand the parameters of our research.’ That would be a way for them to acknowledge they failed in the past to find ways to reach people like me.

Participants desired such acknowledgements to be paired with language explicitly welcoming communities who are historically underrepresented in clinical trials, as echoed by Jasper who said, “let us know that [trials are] open to people of color or LGBT people, you know…come as you are.”

At the community level, participants spent substantial time discussing a desire to see their identities reflected in community‐based clinical trial discussions and expanded education and awareness of trials within communities of color. Mary suggested holding community‐based question‐answer forums. Others mentioned that people of color may lack awareness of clinical trials and have few opportunities to engage in meaningful discussions around trials, as described by Betty who said, “I have not actually come across a discussion of cancer clinical trials anywhere…this information needs to be made public somehow in communities.” Community outreach and engagement could open up pathways for these conversations and facilitate clinical trial participation.

At the interpersonal level, participants sought a supportive professional whose primary goal is to help facilitate enrollment on clinical trials for patients of color. Here, the importance of cultural humility and identity mirroring were put forward as priorities. Simon and others envisioned a professional who would understand “my experience without me having to explain our history of being mistreated, neglected, even abused by people who are White who are healthcare providers.” Indeed, racial and ethnic concordance with clinical trials staff could offer powerful trust‐building opportunities through shared history and mutual understanding.

Participants also cited key characteristics for such a professional, emphasizing the importance of this individual being “highly empathetic”, “knowledgeable”, “communicative,” and “patient.” Notably, participants highlighted that this individual would need to maintain a manageable caseload, as some recalled their inability to receive meaningful Patient Navigator support during treatment due to heavy caseloads. For patient engagement efforts to lay the groundwork for a trusting relationship, participants made it clear that ongoing support and frequent communication are requisites.

## 4 Discussion

Meaningful efforts to better engage communities of color in cancer clinical trials are essential to ensuring that scientific advancements in cancer prevention, treatment, and care equitably benefit all populations. This qualitative study with survivors and caregivers of color provides an in‐depth understanding of factors shaping clinical trial decision‐making, how participants' experiences with just care serve as a model for clinical trials inclusion, how power and inequity manifest in healthcare to limit clinical trials opportunities, and multi‐level approaches for building trust towards clinical trials among communities of color. Below, we build upon these results to offer actionable, anti‐racist strategies for improving racial justice in cancer clinical trials.

All participants were interested in considering clinical trials, and the majority would have been willing to participate if offered, supporting recent studies that have found people of color are as or more likely [43] to join a clinical trial when asked. Despite this, discourse from clinicians and researchers continues to perpetuate the notion that communities of color, and Black survivors in particular, are uninterested in clinical trials [44]. These racially biased beliefs are rooted in assumptions around people of color's resources, the presumption that participation will be uniquely burdensome for people of color, and the idea that previous medical research atrocities have irreparably severed trust. This harmful rhetoric rests on reductionist tropes, disempowers patients, and places crucial decision‐making solely in the hands of healthcare professionals.

Our findings strikingly demonstrate the need for researchers and clinicians to discuss clinical trials with all patients of color, *regardless of eligibility status* for locally available trials. Such conversations would improve patient education and awareness around trials, may facilitate future enrollment, and could empower patients to seek trial opportunities elsewhere. Hiring dedicated staff to equitably approach all patients about clinical trials, optimizing clinical workflows to allow protected time for expanding clinical trial conversations, and increasing trial funding to include designated recruitment and education resources for communities of color are three anti‐racist strategies that could increase capacity for these critical conversations.

Our results suggest that people of color's motivations for participating in clinical trials mirrors those of the predominantly White participants who currently participate—increased chance of survival, altruism, and accessing new treatments. Research teams, industry sponsors, and clinicians should ensure that clinical trial outreach efforts and materials speak to these motivations while ensuring messaging is adaptive and responsive to diverse cultural values, linguistic needs, and intersectional considerations [45, 46]. Involving communities of color in developing and refining such messaging through listening sessions, town halls, community advisory boards, and the inclusion of community members as research partners is an additional anti‐racist strategy to facilitate racial justice [47].

Participants' healthcare experiences, before and after diagnoses, underscore how interactions and relationships with healthcare professionals can serve to facilitate or hinder clinical trial participation. Building trust and a sense of safety between people of color and healthcare professionals is a core component of just care that has positive implications for clinical trial participation [48]. Previous studies demonstrate increased trust [49], comfort [50], and satisfaction [51] among patients who receive care from racially and ethnically concordant providers, a sentiment echoed by our participants. There remains an urgent need to diversify the healthcare and clinical trials workforce by establishing more pathways to engage and sustain students of color in careers in medicine [52]; increasing mentorship opportunities, networking events, and funding to support Principal Investigators of color [53]; and building healthcare and clinical trials site recruitment, hiring, and retention plans through a racial justice lens. However, we caution against oversimplistic conclusions that communities of color *only* trust medical professionals and researchers who reflect their identities. As medicine and health research remain predominantly White‐employed fields [54], White healthcare professionals and clinical researchers can and must engage in racial justice efforts to bridge participation gaps in trials.

Our findings around inequity and trust‐building support Warren et al.'s [55] assertion that “trustworthiness must precede trust.” Participants discussed the layered ways that power manifested within healthcare, from the minimization of their concerns to blatant mistreatment. These are not isolated events, and communities of color understandably distrust systems that have and do perpetuate harm. Thus, healthcare professionals, clinicians, and researchers are called upon to demonstrate an authentic commitment approaching interactions with cultural humility, honoring community and cultural values, explicitly acknowledging and working to remedy the wrongs of research and medical institutions, and exemplifying empathy. Initiatives to support increasing workforce trustworthiness include dedicating person and financial resources for ongoing, comprehensive training focused on unlearning bias and embedding anti‐racist principles into research and clinical practice. These efforts would likely yield more equitable care and mitigate issues such as diagnosis delays resulting from racially biased care encounters.

Participant commentary around agency and choice reveals the importance of ensuring clinical trials happen *with* communities of color, not *to* them. Clinicians can seek to understand what matters most to patients and transparently address whether trials align with those goals. Though clinical trials often have stringent protocols, interweaving principles of patient empowerment [56] in the enrollment process and upholding patient choice where study requirements are flexible can help foster a meaningful balance between regulatory compliance and participants' autonomy, potentially yielding higher enrollments and lower attrition on cancer clinical trials. Providing ample time for patients to consider clinical trial decisions, explicitly encouraging questions, and dedicating space for addressing concerns are essential to embedding agency into the informed consent process. Expanding the availability of decentralized trials could foster patient empowerment by providing communities of color more control over where and how they participate in studies [57, 58]. Taken together, these actions support anti‐racist accountability from healthcare systems and clinical trial staff, which is vital to improving access and enrollment for patients of color.

### 4.1 Limitations

As our participants were predominately cisgender and given the paucity of transgender/nonbinary/two‐spirit patients within clinical trials [59], future research must address gender diversity and add to the growing body of literature [60] surrounding cancer care for LGBTQ+ patients. Most of our participants received treatment within Washington state, limiting the geographic generalizability of our study. Finally, despite offering designated, Spanish‐speaking focus groups and budgeting for a bilingual focus group facilitator, all of our participants spoke English. Future research specifically designed to understand the perspectives of patients whose primary language of communication is not English is critical for developing culturally grounded solutions to opening pathways to clinical trial enrollment for these patients.

## 5 Conclusion

While increasing racial and ethnic diversity in clinical trials has been a longstanding goal among many cancer research teams, coinciding legislative requirements have created a renewed sense of urgency to remedy issues in underrepresentation. Guided by community engagement and anti‐racist research principles, our focus group findings demonstrate that people of color strongly desire clinical trial options and discussions during cancer care and trust‐building is foundational to opening pathways to research participation. We offer concrete steps for increasing racial justice in cancer clinical trials that can serve as a point of reflection and guidance for healthcare professionals, researchers, and clinical trials teams.

## Author Contributions

**Bernadette C. Ray:** investigation (supporting), methodology (supporting), writing – review and editing (supporting). **Chaya M. Pflugeisen:** formal analysis (equal), investigation (equal), methodology (equal), project administration (equal), supervision (equal), writing – original draft (equal), writing – review and editing (equal). **Cynthia Smith:** investigation (supporting), methodology (supporting), project administration (equal). **Deana M. Williams:** conceptualization (lead), formal analysis (equal), funding acquisition (lead), investigation (equal), methodology (equal), project administration (equal), supervision (equal), writing – original draft (equal), writing – review and editing (equal). **De Phillips:** investigation (supporting), methodology (supporting), writing – review and editing (supporting). **Jennifer Slim:** conceptualization (supporting), funding acquisition (supporting), investigation (supporting), methodology (supporting), project administration (supporting), writing – review and editing (supporting). **Keywanna Hardiman:** investigation (supporting), methodology (supporting), writing – review and editing (supporting). **Khadijah Ameen:** investigation (supporting), methodology (supporting), writing – review and editing (equal). **Lizbeth Sanchez:** investigation (supporting), methodology (supporting), writing – review and editing (supporting). **Melissa Foster:** investigation (supporting), methodology (supporting), writing – review and editing (supporting). **Sheree Cooks:** investigation (supporting), methodology (supporting), writing – review and editing (supporting). **Toby K. Chen:** investigation (supporting), methodology (supporting), writing – review and editing (equal).

## Ethics Statement

Ethics approval for this study was obtained by the MultiCare Health System Institutional Review Board.

## Conflicts of Interest

The authors declare no conflicts of interest.

## Data Availability

The data that support the findings of this study are available upon reasonable request from the corresponding author. The data are not publicly available due to privacy restrictions.
